# Cerebral Pulsed Arterial Spin Labeling Perfusion Weighted Imaging Predicts Language and Motor Outcomes in Neonatal Hypoxic-Ischemic Encephalopathy

**DOI:** 10.3389/fped.2020.576489

**Published:** 2020-09-25

**Authors:** Qiang Zheng, Juan Sebastian Martin-Saavedra, Sandra Saade-Lemus, Arastoo Vossough, Giulio Zuccoli, Fabrício Guimarães Gonçalves, Colbey W. Freeman, Minhui Ouyang, Varun Singh, Michael A. Padula, Sara B. Demauro, John Flibotte, Eric C. Eichenwald, John A. Detre, Raymond Wang Sze, Hao Huang, Misun Hwang

**Affiliations:** ^1^Yantai University, Yantai, China; ^2^Children's Hospital of Philadelphia, Philadelphia, PA, United States; ^3^University of Pennsylvania, Philadelphia, PA, United States; ^4^Thomas Jefferson University, Philadelphia, PA, United States

**Keywords:** arterial spin labeling, hypoxic ischemic encephalopathy, neonate, perfusion imaging, perfusion weighted imaging (PWI)

## Abstract

**Rationale and Objectives:** To compare cerebral pulsed arterial spin labeling (PASL) perfusion among controls, hypoxic ischemic encephalopathy (HIE) neonates with normal conventional MRI(HIE/MRI⊕), and HIE neonates with abnormal conventional MRI(HIE/MRI⊖). To create a predictive machine learning model of neurodevelopmental outcomes using cerebral PASL perfusion.

**Materials and Methods:** A total of 73 full-term neonates were evaluated. The cerebral perfusion values were compared by permutation test to identify brain regions with significant perfusion changes among 18 controls, 40 HIE/MRI⊖ patients, and 15 HIE/MRI⊕ patients. A machine learning model was developed to predict neurodevelopmental outcomes using the averaged perfusion in those identified brain regions.

**Results:** Significantly decreased PASL perfusion in HIE/MRI⊖ group, when compared with controls, were found in the anterior corona radiata, caudate, superior frontal gyrus, precentral gyrus. Both significantly increased and decreased cerebral perfusion changes were detected in HIE/MRI⊕ group, when compared with HIE/MRI⊖ group. There were no significant perfusion differences in the cerebellum, brainstem and deep structures of thalamus, putamen, and globus pallidus among the three groups. The machine learning model demonstrated significant correlation (*p* < 0.05) in predicting language(*r* = 0.48) and motor(*r* = 0.57) outcomes in HIE/MRI⊖ patients, and predicting language(*r* = 0.76), and motor(*r* = 0.53) outcomes in an additional group combining HIE/MRI⊖ and HIE/MRI⊕.

**Conclusion:** Perfusion MRI can play an essential role in detecting HIE regardless of findings on conventional MRI and predicting language and motor outcomes in HIE survivors. The perfusion changes may also reveal important insights into the reperfusion response and intrinsic autoregulatory mechanisms. Our results suggest that perfusion imaging may be a useful adjunct to conventional MRI in the evaluation of HIE in clinical practice.

## Introduction

Hypoxic-ischemic encephalopathy (HIE) represents a significant cause of mortality and chronic neurological disability in neonates with heterogeneous short- and long-term outcomes (1). HIE occurs in ~2–3/1,000 births in the developed world (1). Therapeutic hypothermia has shown to reduce morbidity and mortality and improve neurodevelopmental outcomes in infants with moderate to severe HIE (2). Nevertheless, a substantial proportion of affected infants develop neurodevelopmental disorders. Further studies are therefore needed for early detection (3), injury assessment (4), injury pattern evaluation (5), and neurologic outcomes prediction (6).

HIE is currently evaluated with multimodality imaging, including magnetic resonance imaging (MRI) with T1-/T2-weighted imaging (T1WI/T2WI) (7), diffusion-weighted imaging (DWI) (8), diffusion tensor imaging (DTI) (9, 10), magnetic resonance spectroscopy (4), and arterial spin labeling (ASL) (11), and other modalities such as contrast-enhanced ultrasound (12). ASL is a noninvasive perfusion imaging technique that can assess regional cerebral blood flow (CBF) by magnetically labeling inflowing blood. Cerebral perfusion plays an essential role in the diagnosis and prognosis of neonatal HIE (13, 14). Cerebral perfusion changes may provide prognostic information with regard to monitoring reperfusion responses and ongoing injury (13).

Individualized neurodevelopmental outcome predictions in HIE survivors are critically important in assessing patient risk and ensuring proper neuroprotective and adjunctive interventions. Although classification trees have been developed to predict disability or death in infants with HIE (15), accurate prognostication remains challenging due to the broad spectrum of outcomes, ranging from survival with no neurodevelopmental sequelae to death (16).

Imaging may play a role in neurodevelopmental outcome prediction in HIE survivors, which could influence therapeutic decision-making and neuroprotective therapies. However, not all neonatal HIE patients with normal conventional MRI will have normal outcome (17, 18). In this regard, perfusion MRI may be more sensitive than conventional MRI in neonatal HIE. Cerebral perfusion has previously demonstrated value in predicting HIE outcomes, and both hypoperfusion (19) and hyperperfusion (20) may correlate with poor neurodevelopmental outcomes in HIE.

There is a literature gap regarding the evaluation of perfusion alterations in HIE neonates with normal conventional MRI, and the relationship between identified acute perfusion changes in the newborn and neurodevelopmental outcomes. In this study, our first major aim was to employ pulsed ASL (PASL) to explore cerebral perfusion changes in HIE neonates with normal and abnormal conventional brain MRI. We hypothesized that HIE results in cerebral perfusion changes, even in the absence of abnormal findings on conventional MRI. Building on this foundation, the second major aim of this study was to develop a machine learning (ML) model using cerebral PASL perfusion to predict neurodevelopmental outcomes.

## Materials and Methods

### Participants and Data Acquisition

Seventy-three full term neonates were identified as a sample of convenience for a retrospective case-control study following an IRB-approved protocol, between January 2008 and July 2018. A waiver of consent/parental permission, assent and HIPAA authorization has been approved by our IRB. All neonates had T1WI, T2WI, DWI images and PASL of the brain. For clarity, T1WI, T2WI, and DWI sequences will be referred as conventional MRI.

The neonates were divided into three groups: control, HIE/MRI⊖, HIE/MRI⊕. The patients in HIE/MRI⊖ group were with clinical HIE and normal conventional MRI, while the patients from HIE/MRI⊕ group were with clinical HIE and abnormal conventional MRI. There was a fourth additional group combining all HIE patients from the HIE/MRI⊖ and HIE/MRI⊕ groups, called HIE/MRI± group.

The PASL images were acquired with perfusion model of PICORE Q2T from a Siemens 3T scanner using the following parameters: bolus time Tl_1_ = 700 ms, inversion time T_1_ = 1,800 ms, TR/TE = 2,600/14 ms, 14 slices, FOV = 200 × 200 mm, 64 × 64 matrix, voxel size=2.8 × 2.8 × 6.0 mm^3^, flip angle = 90^0^, 45 label/control image pairs. The reason for not using pseudo continuous arterial spin label (PCASL) is that only PASL is clinically available in our retrospective study.

### Control Group Selection

Due to the challenge of finding completely healthy subjects in the context of the inpatient setting and subjects with a clinical indication for neuroimaging, the control cases were selected on the basis of not having any neurological alterations in the recorded neurological examinations (e.g., normal neurological exams, no seizures) during the length of their hospital stay, as well as a conventional brain MRI without neurological abnormalities, and no record of cardiopulmonary arrest, acidemia, or episodes of desaturation requiring intervention with invasive respiratory support. The non-neurological clinical indication for brain MRI was recorded (see Results for all indications).

### HIE Cases Selection

HIE cases were preliminarily identified by clinical indication for brain MRI, including suspected or diagnosed HIE as well as indications potentially associated (e.g., meconium aspiration, cardiopulmonary arrest). Next, all cases were retrospectively reviewed, and only cases categorized as HIE as per the modified Sarnat criteria by the treating physicians were included. At our institution, neonatologists use a modified Sarnat stages scale including: level of consciousness (from stupor/coma to hyperalert/irritable), spontaneous activity, posture, tone, abnormalities of the primitive reflexes (e.g., weak suck or incomplete moro), and autonomic system alterations (e.g., deviated non-reactive pupils, variable heart rate).

### The Bayley-III Scores Acquisition

Developmental assessments were performed using the Bayley Scales of Infant and Toddler Development, 3rd Edition (21), which provides cognitive, language, and motor composite scores at a mean age of 23 months, ranging from 12 to 30 months. Seventeen HIE neonates had 29 outcome data because a proportion of patients had multiple time point outcomes. The outcome data was summarized in Table 1.

**Table 1:** The summarization of the outcome data.

		**Cognitive composite**		**Language composite**		**Motor composite**	
		**>80**	** <80**	**>80**	** <80**	**>80**	** <80**
HIE/MRI⊖ group	Patient number: 13	18	2	12	2	17	0
	Outcome records: 20						
HIE/MRI⊕ group	Patient number: 4	6	3	6	3	7	2
	Outcome records: 9						
HIE/MRI± group	Patient number: 17	24	5	23	5	24	2
	Outcome records: 29						

*^a^ A proportion of patients had multiple time point outcomes, so outcome records > patient number*.

*^b^ If the sum of (>80) and (<80) is smaller than outcome records, that means the data is missing*.

### Image Preprocessing

The ASL data processing toolbox (ASLtbx) was adopted for PASL image preprocessing (22). Motion correction by rigid registration was used to align PASL data to the mean PASL image, temporal-spatial smoothing was performed to prevent noise propagation, PASL perfusion difference images were computed by subtracting the time-averaged signal intensities of control and label images, and outlier cleaning was applied after perfusion subtraction to remove outlier PASL acquisition time points (23). The CBF map for PASL data was calculated by applying the single-compartment mode (24).

(1) $$\begin{array}{l} CBF=\frac{6000\cdot \lambda \cdot \left(SI_{control}-SI_{label}\right)\cdot e^{\frac{TI}{T_{1b}}}}{2\cdot \alpha \cdot TI_{1}\cdot M_{0b}}\;\left[ml/100g/\min\right] \end{array}$$

where *SI*_*control*_ and *SI*_*label*_ were the time-averaged signal intensities of control and label images, respectively. the blood-brain partition coefficient λ = 1.10 *mL*/*g* for neonates (25, 26), *T*_1*b*_ = 1, 825*ms* for neonates was used for the longitudinal relaxation time of blood at 3.0 T MRI (27), labeling efficiency α = 0.98 for PASL (24), the bolus time *TI*_1_ = 700 *ms*, and inversion time *TI* = 1, 800 *ms*. The factor of 6,000 converts the units from ml/g/s to the customary ml/100 g/min (28).

*M*_0*b*_ is the relaxed equilibrium magnetization of the arterial blood and calculated from the proton density-weighted image *M*_0_ with fully relaxed blood spins as follows:

(2) $$\begin{array}{l} M_{0b}=R_{i}\cdot M_{0i}\cdot exp\left(TE/T_{2i}-TE/T_{2b}\right) \end{array}$$

where *T*_2*b*_ = 191*ms* for neonates (27). In our study, the *M*_0*b*_ was calculated on cerebrospinal fluid (CSF), white matter (WM), and gray matter (GM) separately with *i* = *CSF, WM or GM* in Equation (2). Specifically, *R*_*CSF*_ = 0.87, *R*_*WM*_ = 1.19, *R*_*GM*_ = 0.98 (29) were the signal ratios of the tissue type used to blood from a proton density-weighted image, *T*_2*CSF*_ = 250*ms*, *T*_2*WM*_ = 222*ms* and *T*_2*GM*_ = 143*ms* (30) were used for neonates. To calculate *M*_0*CSF*_, *M*_0*WM*_, and *M*_0*GM*_, we aligned the T2 image to *M*_0_ space, and segmented the image into the CSF, WM and GM by thresholds 0.95 and 0.75. *M*_0*i*_ was the average signal intensity of CSF, WM or GM in *M*_0_.

### Statistical Analysis for Group Comparison

**Figure 1:**
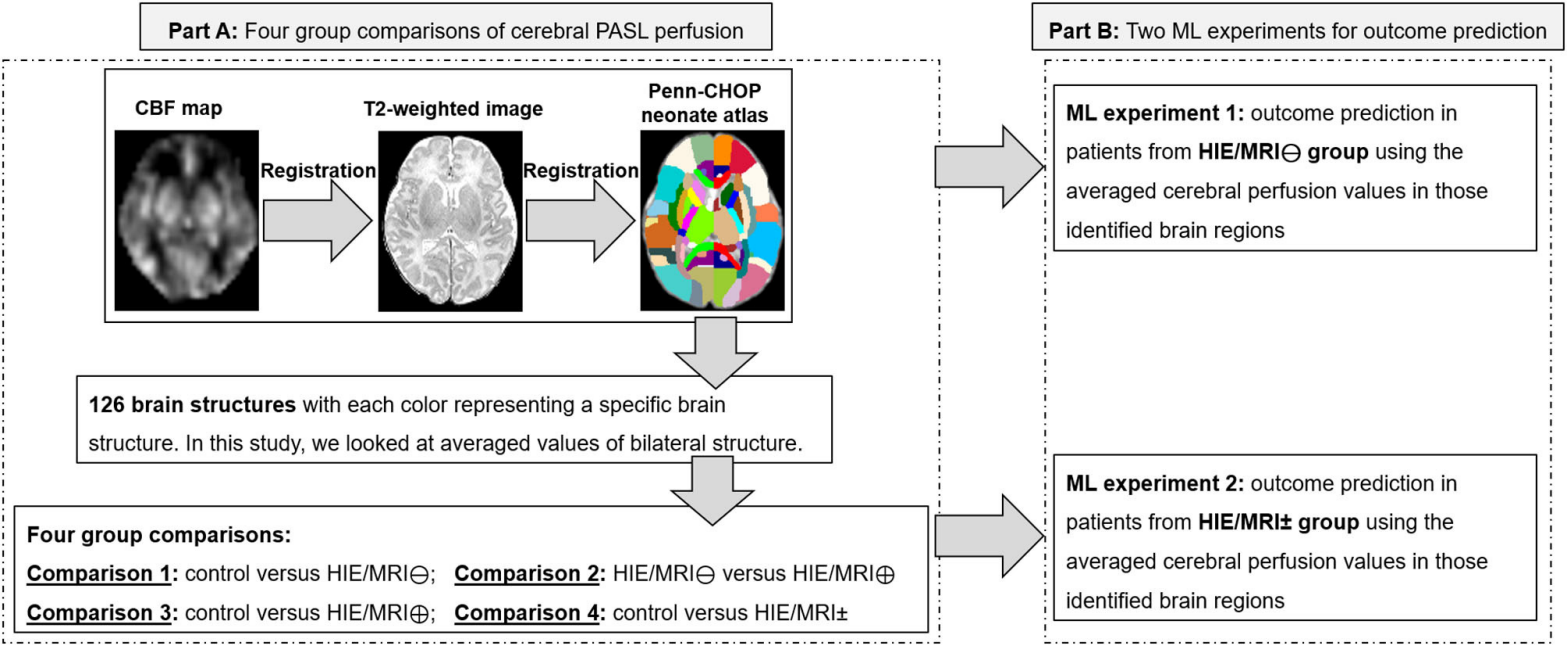
Flow chart of the processing method. Part A: Four group comparisons of cerebral PASL perfusion. Part B: Two ML experiments for outcome prediction. In Part B, the ML experiment was not performed in patients from the HIE/MRI⊕ group separately due to limited patients having long-term outcomes in this group. ML, machine learning.

In part A of this study (Figure 1), a Penn-CHOP neonatal brain atlas (31) was adopted to identify brain regions by statistical comparison among the control, HIE/MRI⊖, HIE/MRI⊕, and HIE/MRI± groups. Four comparisons between groups were performed: (1) control vs. HIE/MRI⊖; (2) HIE/MRI⊕ vs. HIE/MRI⊕; (3) control vs. HIE/MRI⊕; (4) control vs. HIE/MRI±. CBF maps were aligned to the atlas space by the bridge of the subject native T2 space. Linear regression was performed to eliminate the effect of age and gender on the PASL data before statistical group comparison.

The averaged perfusion values in segmented brain structures were compared by permutation test to identify brain regions with statistically significant perfusion changes. The regional CBF map of each subject was normalized to their own averaged whole brain CBF value before group comparison. The normalization makes the CBF values more comparable across different subjects due to a big variation of the absolute CBF values. Therefore, the CBF values being quantified in this study are relative perfusion, not absolute perfusion.

### Machine-Learning-Assisted Outcome Prediction

In part B of this study (Figure 1), linear regression was used to predict the cognitive, language, and motor outcomes by leave-one-out cross-validation with feature dimensionality reduction by principal component analysis (PCA), where the optimal feature dimensionality was determined by grid search.

Two ML experiments were performed for predicting outcomes in patients from HIE/MRI⊖ and HIE/MRI± groups (Part B in Figure 1), respectively. The ML experiment was not performed in patients from the HIE/MRI⊕ group separately due to limited number of patients having long-term outcomes. The features fed into each ML experiment were slightly different when used for predicting outcomes.

For the experiment 1 (performed in the HIE/MRI⊖ group), the feature was a vector of averaged perfusion values from brain regions with significant perfusion changes obtained by comparison 1 (Part A in Figure 1).

For the experiment 2 (performed in the HIE/MRI ± group), the feature was a concatenated vector of averaged perfusion values from brain regions with significant intra- and inter- perfusion differences between the four groups. The intra- and inter perfusion differences were obtained by comparisons 2 and 4, respectively (Part A in Figure 1).

## Results

### Subjects' Groups

#### Controls

A total of 18 neonates (10 males and eight females, age 16 ± 7.4 days at MRI, full term) were included as controls. The indications for brain MRI in these subjects were the following: facial hemangioma, assessment of congenital diaphragmatic hernia (in two cases), periodic breathing, subjective cyanotic episodes as reported by the caretakers (in four cases), poor feeding (in two cases), osteogenesis imperfecta, cardiac mass, cervical lymphangioma, follow-up of herpes simplex virus infection, follow-up of subdural hygromas, and periauricular scalp arteriovenous malformation.

#### HIE Cases

In the HIE/MRI⊖ group, there were 40 neonates (25 males and 15 females, age 8.7 ± 6.1 days at MRI, full term) with clinical HIE and normal conventional MRI. Thirty-two neonates underwent hypothermia therapy in this group. Besides, the HIE/MRI⊕ group consisted of nine mild cases, 11 moderate cases, 0 severe case, 20 cases with unknown severity.

In the HIE/MRI⊖ group, there were 15 neonates (six males and nine females, age 6.4 ± 4.5 days at MRI, full term) with clinical HIE and abnormal conventional MRI. Ten neonates underwent hypothermia therapy in this group. Besides, the HIE/MRI⊕ group consisted of one mild cases, five moderate cases, six severe cases, three cases with unknown severity.

The additional HIE/MRI± group is the combination of HIE/MRI⊖ and HIE/MRI⊕ groups.

### Cerebral ASL Perfusion Changes in Both HIE/MRI⊖ and HIE/MRI⊕ Groups

Significantly decreased perfusion was found in the HIE/MRI⊖ group, when compared with controls (Table 2A). Decreased values were seen primarily in the anterior corona radiata, caudate, superior fronto-occipital fasciculus, superior frontal gyrus, precentral gyrus.

**Table 2:** Brain regions with significant perfusion differences between control and HIE/MRI⊖ groups, and between HIE/MRI⊖ and HIE/MRI⊕ groups.

**Brain regions**	**Normalized CBF (mean** **±** **sd)**		***p*-value**
**A: Control vs. HIE/MRI⊖**	**Control**	**HIE/MRI⊖**	**(*p* < 0.05)**
A.nterior corona radiate	0.96 ± 0.54	0.74 ± 0.29^#^	0.015
Caudate	1.31 ± 0.34	1.09 ± 0.41^#^	0.033
Superior fronto-occipital fasciculus	0.99 ± 0.44	0.86 ± 0.36^#^	0.046
Superior frontal gyrus	1.13 ± 0.34	1.02 ± 0.21^#^	0.043
Precentral gyrus	1.43 ± 0.25	1.38 ± 0.18^#^	0.009
**B: HIE/MRI⊖** **vs. HIE/MRI⊕**	**HIE/MRI⊖**	**HIE/MRI⊕**	**(*****p*** **<** **0.05)**
Cingulum hippocampal part	2.33 ± 0.60	1.98 ± 0.43^#^	0.015
Uncinate fasciculus	1.76 ± 0.65	1.41 ± 0.48^#^	0.032
Gyrus rectus	1.52 ± 0.52	1.15 ± 0.25^#^	0.034
Precentral gyrus	1.38 ± 0.18	1.58 ± 0.41^*^	0.028
Postcentral gyrus	1.40 ± 0.21	1.60 ± 0.36^*^	0.016
Superior parietal gyrus	1.01 ± 0.32	1.24 ± 0.32^*^	0.014
Precuneus	1.15 ± 0.42	1.44 ± 0.34^*^	0.016
Parahippocampal gyrus	2.22 ± 0.47	1.86 ± 0.45^#^	0.009
Superior occipital gyrus	1.02 ± 0.47	1.36 ± 0.58^*^	0.020
Middle occipital gyrus	1.04 ± 0.32	1.44 ± 0.48^*^	<0.001
Cuneus	1.43 ± 0.36	1.85 ± 0.54^*^	0.003
Hippocampus	2.09 ± 0.41	1.77 ± 0.40^#^	0.008

# *Decreased perfusion*;

* *increased perfusion*.

*Normalized CBF values of different brain regions and p-values for all groups*.

Both significantly increased and decreased PASL perfusion changes were detected in the HIE/MRI⊖ group, when compared with the HIE/MRI⊕ group. Decreased PASL perfusion values were seen primarily in cingulum hippocampal part, uncinate fasciculus, gyrus rectus, parahippocampal gyrus, and hippocampus, while increased PASL perfusion values were seen primarily in the precentral gyrus, postcentral gyrus, superior parietal gyrus, superior occipital gyrus, middle occipital gyrus, and cuneus.

Further comparisons were performed between control and HIE/MRI⊖ groups (Table 3A) and between control and HIE/MRI± (Table 3B). Both significantly increased and decreased PASL perfusion changes were also detected in the HIE/MRI⊕ group, but only decreased PASL perfusion changes were detected in the HIE/MRI± group. The brain structures with significant perfusion changes have big overlaps between Tables 3A, 2B, and between Tables 2A, 3B.

**Table 3:** Brain regions with significant perfusion differences between control and HIE/MRI⊖ groups, and between control and HIE/MRI ± groups.

**Brain regions**	**Normalized CBF (mean** **±** **sd)**		***p*-value**
**A: Control vs. HIE/MRI⊕**	**Control**	**HIE/MRI⊕**	**(*p* < 0.05)**
Corpus callosum (body)	1.42 ± 0.24	1.27 ± 0.24^#^	0.017
Tapetum	1.08 ± 0.31	0.81 ± 0.33^#^	0.035
Gyrus rectus	1.52 ± 0.45	1.15 ± 0.25^#^	0.016
Superior parietal gyrus	1.05 ± 0.27	1.24 ± 0.32*	0.017
Precuneus	1.16 ± 0.36	1.44 ± 0.34*	0.008
Superior occipital gyrus	1.03 ± 0.43	1.36 ± 0.58*	0.020
Middle occipital gyrus	1.12 ± 0.40	1.44 ± 0.48*	0.008
Cuneus	1.42 ± 0.49	1.85 ± 0.54*	0.023
**B: Control vs. HIE/MRI±**	**Control**	**HIE reading** **±**	**(*****p*** **<** **0·05)**
Anterior corona radiate	0.96 ± 0.54	0.73 ± 0.34^#^	0.020
Caudate	1.31 ± 0.34	1.08 ± 0.44^#^	0.026
Superior frontal gyrus	1.13 ± 0.34	1.00 ± 0.22^#^	0.017

# Decreased perfusion; *increased perfusion.

*Normalized CBF values of different brain regions and p-values for all groups*.

There were no significant perfusion differences in the cerebellum, brainstem and deep structures such as the thalamus, putamen, and the globus pallidus among the control, HIE/MRI⊖ group, and HIE/MRI⊕ group.

### Cerebral PASL Perfusion Predicts Language and Motor Outcomes in Both HIE/MRI⊖ and HIE/MRI± Groups

**Figure 2:**
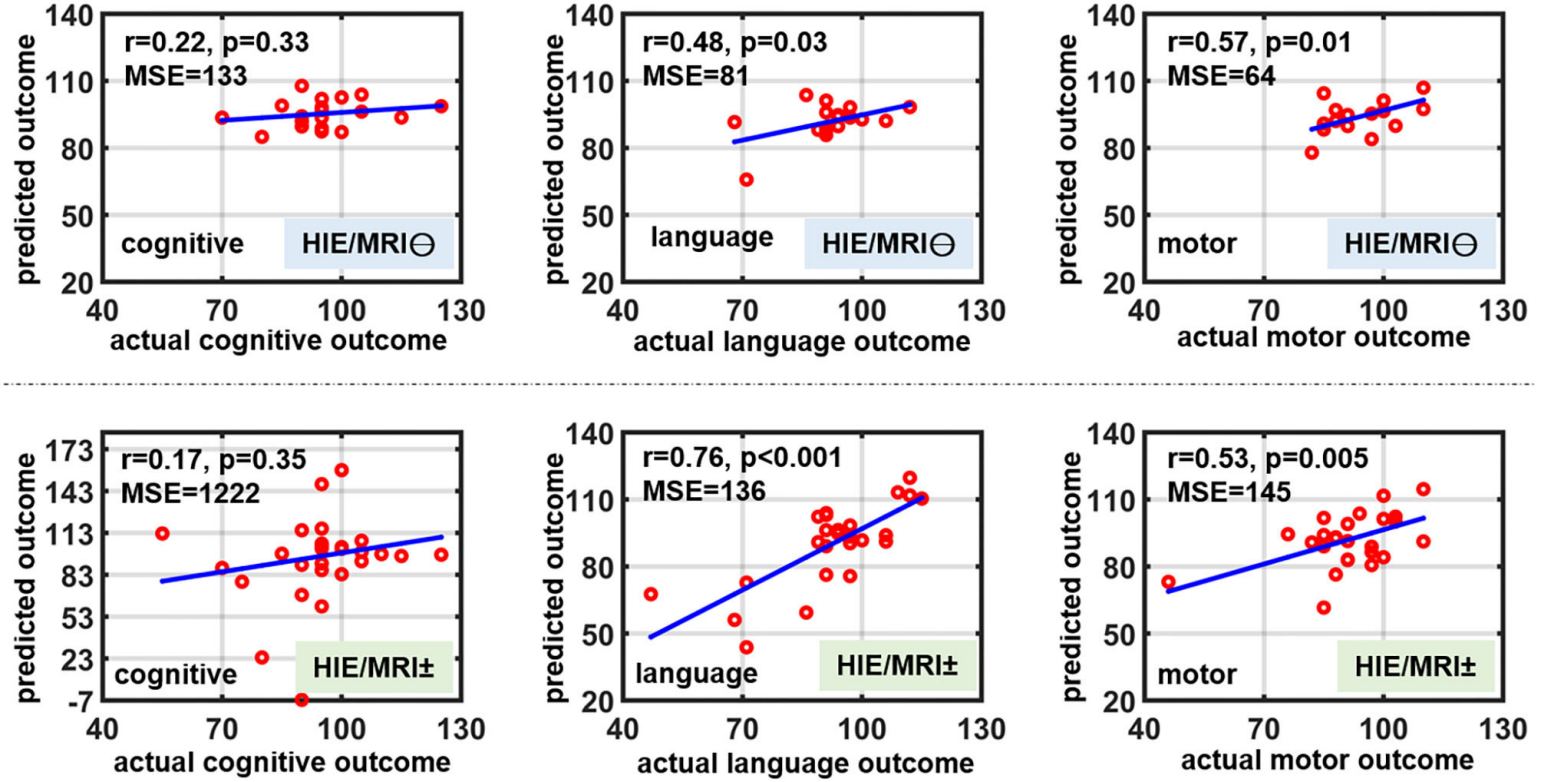
Outcome prediction performance in HIE/MRI⊖ group (first line) and HIE/MRI ± group (second line). In the both groups, the predicted outcomes by machine learning model are significantly correlated with language and motor outcomes, but not with cognitive outcome.

Following ML experiment 1 (Part B in Figure 1, HIE/MRI⊖ group) and using the feature vector of averaged perfusion values from brain regions with significant perfusion changes obtained by comparison 1 (Part A in Figure 1), the ML-assisted model demonstrated significant positive correlation values in predicting language (*r* = 0.48, *p* = 0.03) and motor (*r* = 0.57, *p* = 0.01) outcomes in the HIE/MRI⊖ group (*p* < 0.05), but not significant correlation in predicting cognitive outcome (*p* = 0.33). (First row in Figure 2).

Following ML experiment 2 (Part B in Figure 1, HIE/MRI± group) and using the concatenated feature vector of averaged perfusion values from brain regions with significant intra- and inter- perfusion differences obtained by comparisons 2 and 4 (Part A in Figure 1), the ML-assisted model demonstrated significant positive correlation values in predicting language (*r* = 0.76, *p* < 0.001) and motor (*r* = 0.53, *p* = 0.005) outcomes in the HIE/MRI± group (*p* < 0.05), but not significant correlation in predicting cognitive outcome (*p* = 0.35). (Second row in Figure 2).

## Discussion

HIE is a significant cause of morbidity and mortality in neonates (1). A considerable proportion of neonatal HIE survivors develop cognitive, language, and motor disorders. Cerebral perfusion has previously demonstrated value in predicting HIE outcomes, and both hypoperfusion (19) and hyperperfusion (20) may correlate with poor neurodevelopmental outcomes in HIE. ASL perfusion may play a role in the prediction of neurodevelopmental outcome in HIE survivors, which could influence therapeutic decision-making and implementation of neuroprotective therapies.

In this study, cerebral ASL perfusion changes were detected in different brain regions in HIE patients, regardless of findings on conventional MRI. Specifically, decreased cerebral ASL perfusions were detected in HIE/MRI⊖ group when compared with controls. The clinical significance of cerebral ASL perfusion changes is further evidenced by the predictive nature of these values to stratify language and motor outcomes in HIE patients, and those identified brain structures have been associated with language and motor functions. For instance, the precentral gyrus is the anatomical location of the primary motor cortex (32). Caudate nuclei, constituting the subcortical nuclei of basal ganglia, is responsible for motor control (33) and language (34). Superior frontal gyrus (35) and corona radiata (36) are also related to complex motor functions. These are evidences that ASL perfusion may be more sensitive than conventional MRI, including DWI, in the neuroimaging evaluation of neonatal HIE. Therefore, clinicians and radiologists should not rely solely on conventional MRI sequences in the neuroimaging evaluation of HIE.

There were no significant perfusion differences in the cerebellum, brainstem and deep structures such as the thalamus, putamen, and the globus pallidus among the control, HIE/MRI⊖ group, and HIE/MRI⊕ group. This may serve as an evidence that the intrinsic autoregulatory mechanisms are able to maintain perfusion to these areas of the brain and spare them from injury in mild to moderate hypoxic-ischemic insults (37) which constituted the majority of our retrospective cohort. Experiments performed in animal models have demonstrated that episodes of prolonged fetal hypoxia result in shunting of blood to vital brain structures, such as the brainstem, thalami, basal ganglia, and cerebellum, at the expense of less metabolically active structures, namely, the cerebral cortex and white matter (38). It should be noted, however, that prolonged, severe hypoxic ischemic event leads to permanent injury to the basal ganglia and thalami which is correlated with poor outcomes (39).

Both significantly increased and decreased PASL perfusion changes were detected in the HIE/MRI⊕ group, when compared with the HIE/MRI⊖ group or controls. Cortical structures are demonstrating increased perfusion in HIE/MRI⊕ group than HIE/MRI⊖ patients or controls with the exception of limbic structures including hippocampus, parahippocampal gyrus, cingulum hippocampal part and corpus callosum. The reason for cortical hyperperfusion could be due to ongoing reperfusion response and/or impaired autoregulation. With regard to decreased perfusion in the limbic system, it would be worthwhile to explore whether this represents progression to permanent injury given that these regions have been shown to be vulnerable to hypoxic ischemic insult (40, 41).

**Figure 3:**
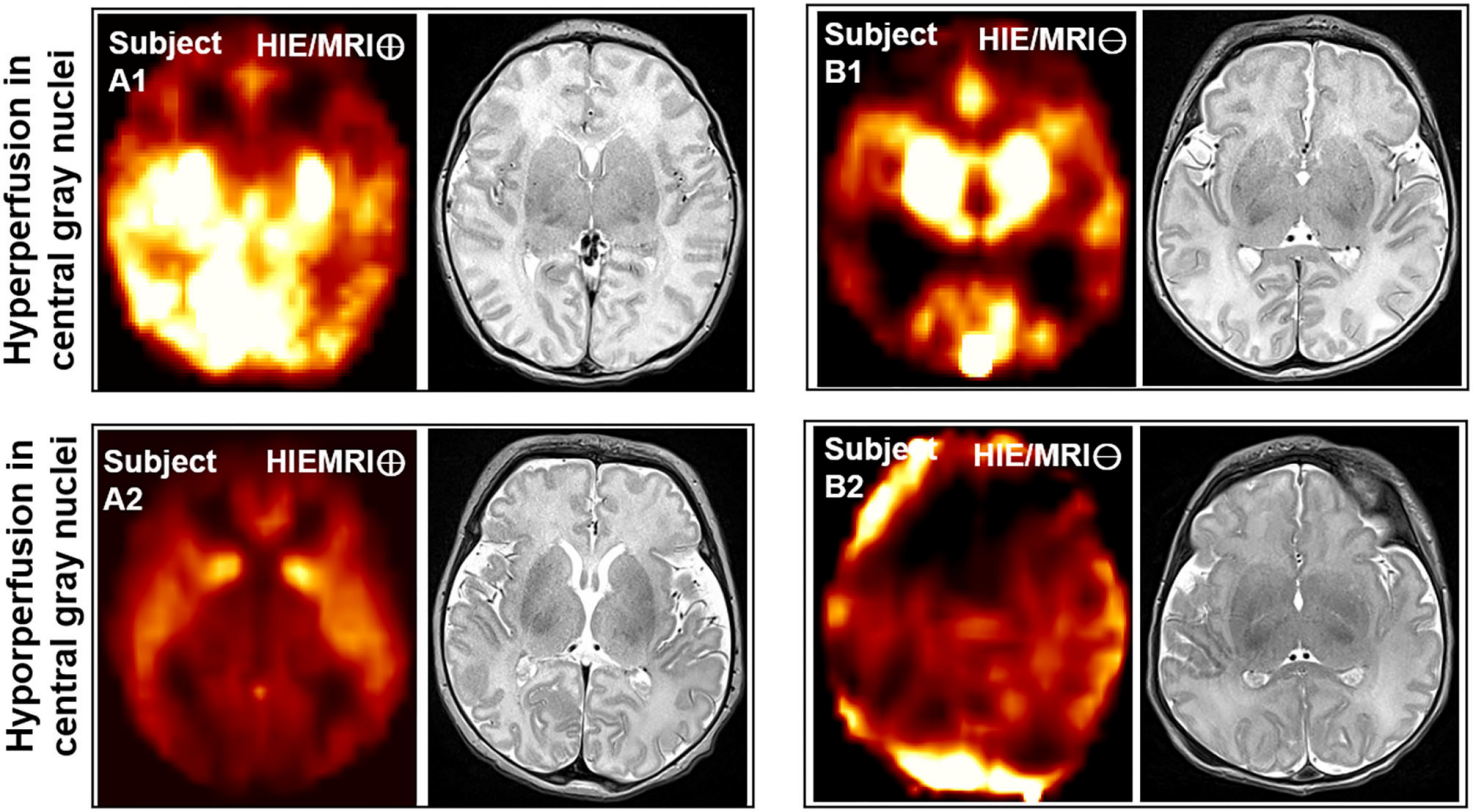
Axial views of CBF map and T2WI image comparison of different HIE patients with good and poor outcome in the 1st and 2nd rows. Subject A1 is from HIE/MRI⊕ group, having hyperperfusion in those vital brain structures and Bayley-III scores of cognitive = 105(>80), language = 112(>80), and motor = 97(>80); Subject A2 is from HIE/MRI⊖ group, having hypoperfusion in those vital brain structures and Bayley-III scores of cognitive = 75(<80), language = 71(<80), and motor = 76(<80); Subject B1 is from HIE/MRI⊕ group, having hyperperfusion in those vital brain structures and Bayley-III scores of cognitive = 105(>80), language = 91(>80), and motor = 97(>80); Subject B2 is from HIE/MRI⊖ group, having hypoperfusion in those vital brain structures and Bayley-III scores of cognitive = 55(<80), language = 47(<80), and motor = 46(<80). The vital brain structures refer to cerebellum, brainstem and deep structures such as the thalamus, putamen, and the globus pallidus.

In the present study, we also found both hyperperfusion and hypoperfusion in those vital brain structures of cerebellum, brainstem and deep structures in both HIE/MRI⊖ and HIE/MRI⊕ groups. As shown in Figure 3, the patients with hypoperfusion in those vital brain structures had Bayley-III scores lower than 80 implying poor long-term outcomes, while the patients with hyperperfusion in those regions had good long-term outcomes with Bayley-III scores >80. This further demonstrated that hypoperfusion in those vital brain structures might progress to irreversible brain injury (42) and lead to poor long-term outcome.

A few limitations existed in the present study. (1) Accessing completely healthy subjects is challenging in the context of an inpatient and sick population, thus, subjects with other non-neurological abnormalities were included as controls. (2) The developmental assessments were carried out on a small data size with Bayley-III score in a relatively wide range of age in composite scores acquisition. (3) The selection of HIE cases was done on the basis of clinical diagnosis in real time by their treating physicians, using a fairly standardized clinical scale in place at our institution (as described in the Methods section) that nonetheless may be subjective and introduces heterogeneity in the HIE cases. (4) ASL data of neonatal brain is low resolution, which may raise questions of the validity of perfusion values in small anatomic structures. In this regard, we believe that in the future, the robustness can further be improved with additional data.

Additionally, in the interpretation of our results, we acknowledge that the timing and degree of reperfusion is dependent on a wide spectrum of factors including selective vulnerability of brain regions, the severity of injury incurred, the extent of reperfusion response, the susceptibility of different brain regions to reperfusion injury, the timing of injury, the timing of imaging, and the presence or absence of subclinical seizures. For instance, brain MRI performed closer to the termination of therapeutic hypothermia may demonstrate increased perfusion as a sequelae of restorative perfusion. It would be interesting to assess in this regard whether the extent of reperfusion has implications on later outcomes, and whether this is dependent on gender, age, injury type or duration, and/or injury severity. Interpretation of brain perfusion also has inherent challenges, as it is difficult to discern restorative, beneficial reperfusion from reperfusion injury wherein marked metabolism perfusion uncoupling can result in high levels of reactive oxygen species and permanent brain damage. Despite the complexity of brain perfusion, our results show that it is indeed an important imaging marker for outcomes in HIE and that more carefully designed prospective studies are warranted to prospectively evaluate the spatiotemporal dynamics of brain perfusion.

In summary, we demonstrate that cerebral perfusion changes can be detected in HIE patients, including those with normal conventional MRI, highlighting the value of perfusion imaging in evaluating suspected HIE. The perfusion changes may reveal important insights into how individual's autoregulatory and reperfusion response to hypoxic insult influences outcomes. Moreover, these perfusion values significantly correlate with language and motor outcomes in HIE patients with normal and abnormal conventional MRI. Therefore, perfusion imaging is a promising tool for early risk stratification and prediction of language and motor outcomes in HIE survivors and may be a useful adjunct to conventional MRI in the evaluation of HIE in clinical practice.

## Data Availability Statement

The raw data supporting the conclusions of this article will be made available by the authors, without undue reservation.

## Ethics Statement

The studies involving human participants were reviewed and approved by Children's Hospital of Philadelphia. Written informed consent to participate in this study was waived.

## Author Contributions

All authors have made substantial contributions to all of the following: (1) the conception and design of the study, or acquisition of data, or analysis and interpretation of data, (2) drafting the article or revising it critically for important intellectual content, (3) final approval of the version to be submitted.

## Conflict of Interest

The authors declare that the research was conducted in the absence of any commercial or financial relationships that could be construed as a potential conflict of interest.
